# Recurrent giant retroperitoneal liposarcoma: A case report

**DOI:** 10.1097/MD.0000000000050815

**Published:** 2026-10-02

**Authors:** Meng-wei Tu, Song Zhu, Jian-bo Zhang

**Affiliations:** a Department of Gastrointestinal, Thyroid and Breast Surgery, Chongqing Emergency Medical Center, Chongqing, China; b Department of Thyroid, Breast and Gastrointestinal Surgery, The First People’s Hospital of Liangshan Yi Autonomous Prefecture, Xichang, Sichuan, China; c Department of Gastrointestinal and Anorectal Surgery, The Second Affiliated Hospital of Chongqing Medical University (Chongqing Medical University), Chongqing, China.

**Keywords:** giant tumor, myxoid liposarcoma, retroperitoneal liposarcoma, soft tissue sarcoma, tumor recurrence

## Abstract

**Rationale:**

This report describes an exceptionally large recurrent tumor with a long-standing and recurrent clinical course, thereby adding to the limited literature on giant myxoid liposarcoma.

**Patient concerns:**

A 48-year-old male with a 13-year history of recurrent liposarcoma presented with progressive abdominal distension and marked abdominal enlargement over 4 months. Imaging demonstrated a massive abdominopelvic mass measuring 352 mm in maximum diameter.

**Diagnoses:**

Histopathological examinations from multiple prior resections had consistently confirmed recurrent myxoid liposarcoma.

**Interventions:**

During the current admission, the patient underwent complete resection of a giant retroperitoneal tumor, with pathology again verifying myxoid liposarcoma. No adjuvant chemotherapy or radiotherapy was administered.

**Outcomes:**

At 18-month follow-up, the patient developed multiple abdominopelvic metastases, indicating the aggressive biological behavior and high recurrence risk of retroperitoneal myxoid liposarcoma.

**Lessons:**

This case describes an exceptionally large recurrent retroperitoneal liposarcoma measuring 43 × 32 × 23 cm and weighing approximately 20 kg, with a prolonged 13-year clinical course characterized by multiple recurrences and repeated surgical resections. It demonstrates that even an extremely large recurrent tumor may remain amenable to surgical resection following comprehensive imaging evaluation and multidisciplinary planning, with appropriate preparation for possible adjacent-organ resection. Nevertheless, the development of multifocal abdominopelvic recurrence 18 months after surgery underscores the persistent risk of recurrence and the need for structured, long-term postoperative surveillance. This case contributes to the limited literature by illustrating both the feasibility and the inherent limitations of repeated surgical management for giant recurrent retroperitoneal liposarcoma.

## 1. Introduction

Liposarcoma is a malignant tumor arising from primitive mesenchymal cells differentiating into adipocytes, most commonly in the extremities and retroperitoneum.^[1]^ Retroperitoneal liposarcoma (RPLS) is rare, accounting for 0.07% to 0.2% of all tumors and 10% to 15% of soft tissue sarcomas, with a peak incidence at 40 to 60 years and similar rates in both sexes.^[2]^ The World Health Organization classifies RPLS into 5 subtypes: well-differentiated, dedifferentiated, myxoid, pleomorphic, and myxoid-pleomorphic liposarcoma. Giant liposarcomas are defined as tumors ≥30 cm in diameter or ≥20 kg in weight, and despite their malignancy, only about 10% metastasize hematogenously.

RPLS is often asymptomatic until reaching >10 cm, with early signs including abdominal distension, early satiety, and abdominal or back pain.^[3]^ Contrast-enhanced computed tomography (CT) is the imaging modality of choice, providing critical information on tumor extent and surrounding tissue involvement.^[4]^ Percutaneous biopsy is generally discouraged due to risks of hemorrhage and tumor seeding.^[5]^ Histopathology remains the diagnostic gold standard and is essential for grading and assessing tumor behavior. When imaging is typical and the tumor is deemed resectable, preoperative biopsy may be omitted to reduce the risk of recurrence and metastasis.^[6]^

Due to the limited efficacy of chemotherapy and radiotherapy, complete surgical resection remains the gold standard for both primary and recurrent RPLS.^[7,8]^ Most soft tissue sarcomas are surrounded by a pseudocapsule of cellular debris, inflammatory cells, and residual tumor tissue, reflecting their invasive nature and the ongoing risk of recurrence and metastasis after surgery.^[9]^ Notably, well-differentiated liposarcomas generally exhibit a lower grade and metastatic potential, whereas myxoid subtypes are a higher grade with greater metastatic propensity.^[10]^

## 2. Case presentation

A 48-year-old male with a history of hypertension and diabetes presented with recurrent lumbar liposarcoma. Thirteen years prior, he underwent initial resection, with pathology confirming liposarcoma. Over the following 13 years, he experienced 3 recurrences, undergoing 4 surgeries in total. After the second resection, he received 2 cycles of chemotherapy and 1 month of radiotherapy. Post-third surgery, repeated debridements were performed for infection, with histology showing ulceration but no tumor recurrence.

Four months before admission, the patient developed progressive abdominal distension. Over the following 2 months, his body weight increased from approximately 80 kg to 95 kg. Physical examination revealed marked abdominal distension; however, serial measurements of abdominal circumference and complete previous imaging records documenting tumor progression were unavailable due to the prolonged disease course and multiple previous operations performed over 13 years. Nevertheless, the substantial increase in body weight and the massive tumor burden demonstrated by current imaging indicated a significant disease burden at presentation.

Contrast-enhanced CT of the abdomen demonstrated a massive, irregular intra-abdominal and pelvic mass measuring approximately 352 × 342 × 332 mm, predominantly of low and fat density, with relatively clear margins and marked compression and displacement of adjacent organs and vessels (Fig. 1). Based on the patient’s history of recurrent liposarcoma, the histopathological findings from previous surgical resections, and the current CT features of a giant fat-containing retroperitoneal mass, recurrent RPLS was considered the leading preoperative diagnosis. Retroperitoneal lipoma was included in the differential diagnosis because it may also present as a predominantly fat-containing mass. However, this possibility was considered less likely given the exceptionally large tumor size, irregular morphology, and heterogeneous internal density, together with the patient’s well-documented history of liposarcoma. No separate preoperative biopsy was performed during the current disease course because the patient had a well-established pathological diagnosis from previous resections, and additional biopsy was considered unlikely to alter the planned treatment strategy. Nevertheless, imaging alone could not reliably determine the histological subtype, and the definitive diagnosis was established by postoperative histopathological examination. Following multidisciplinary evaluation, the surgical team decided to proceed with tumor resection.

**Figure 1.**
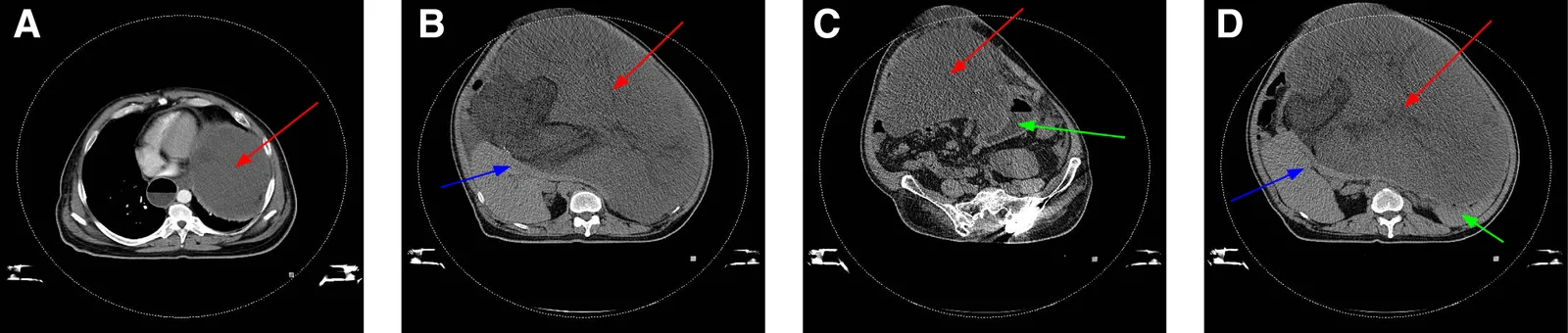
The computed tomography findings at different levels of this patient, with the tumor shown by the red arrows (A, B, C, and D); the liver shown by the blue arrow, which can be seen to be compressed and pushed toward the posterior abdomen (B, D); and the stomach shown by the green arrow, which can be seen to be compressed (C, D).

During open laparotomy, the tumor was found to occupy nearly the entire abdominal cavity, originating from the retroperitoneum and displacing the liver, spleen, stomach, and intestines posteriorly. The tumor adhered locally to the diaphragm, necessitating partial diaphragmatic resection, while other abdominal organs were preserved. Postoperatively, the patient’s abdomen was notably flattened. The resected mass measured approximately 430 × 320 × 230 mm and weighed about 20 kg (Fig. 2). Histopathological examination confirmed a myxoid liposarcoma (Fig. 3). At discharge, the patient’s body weight had decreased to 77 kg, with a body mass index of 28.2 kg/m^2^.

**Figure 2.**
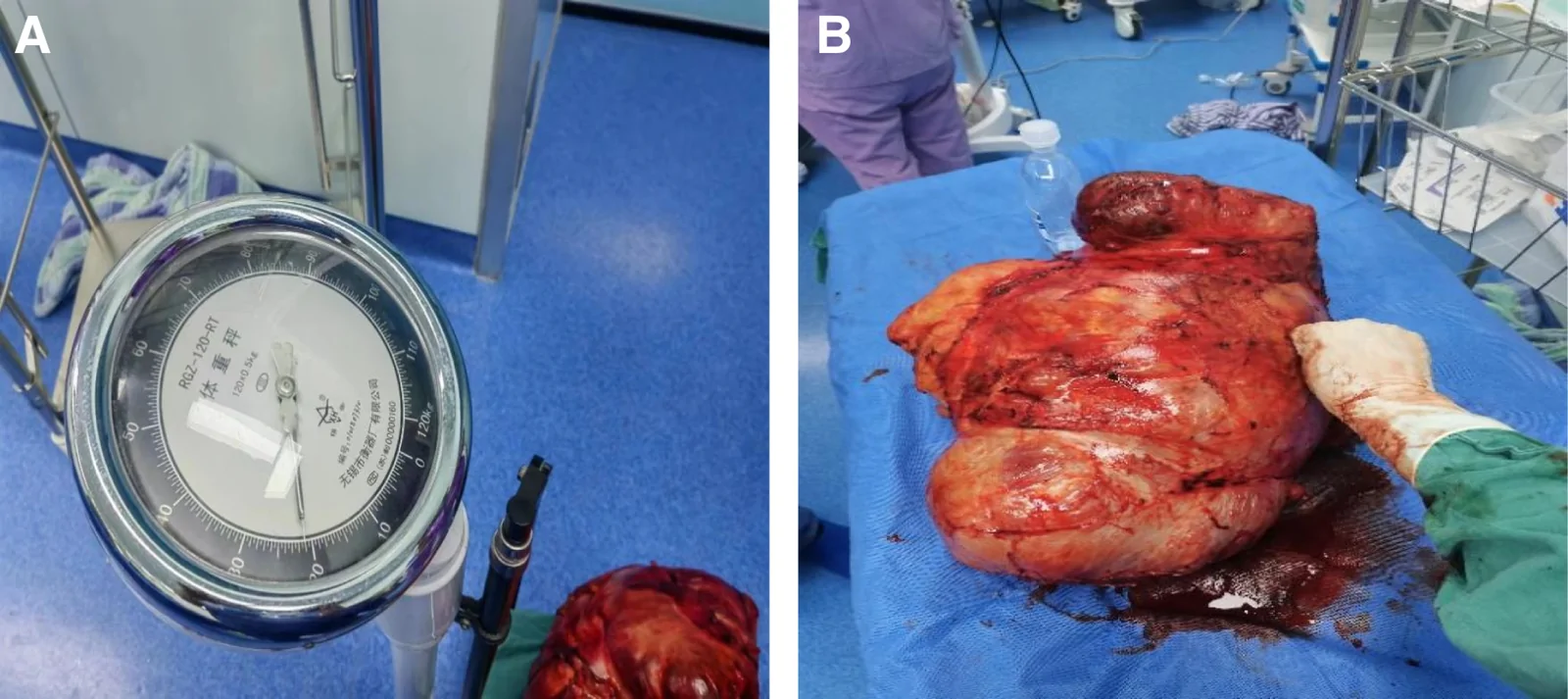
(A) A completely resected tumor with an irregular shape after surgery. The size of this tumor was measured as 43 × 32 × 23 cm. (B) Weighing of this tumor on an electronic scale. The needle of this electronic scale points to 2 kg without the tumor, and the needle shows 22 kg after this liposarcoma was put on the scale.

**Figure 3.**
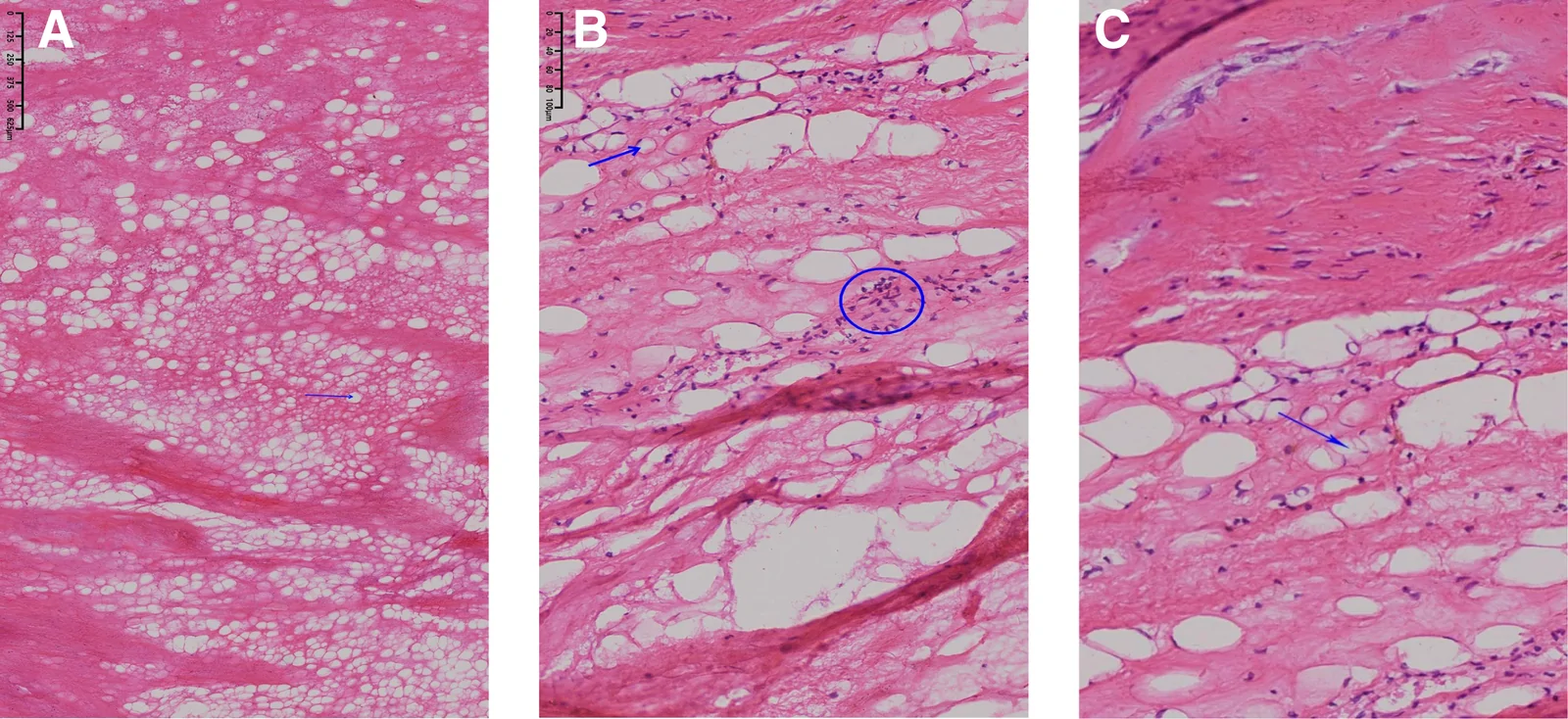
(A) A section of a pathological specimen microscopically at 40×. A large number of adipocytes are seen. The arrow points to a normal adipocyte in which lipid droplets are seen. (B) A section of a pathological specimen microscopically at 200×. The arrow points to an imprinted adipoblast, and a large number of normal mesenchymal cells are seen in the area framed by the circle. (C) A section of a pathological specimen at 200× with a rich mucus-like background, and mucus is seen at the point where the arrow points to an aggregation.

The patient did not undergo regular postoperative follow-up. Eighteen months after surgery, he presented with abdominal distension and decreased appetite. Contrast-enhanced CT of the entire abdomen revealed multiple masses in the abdominopelvic cavity, with marked displacement of adjacent organs and vessels, confirming tumor recurrence (Fig. 4). After evaluation by the surgical team, the patient underwent repeat tumor resection. Intraoperatively, multiple abdominopelvic masses were observed, the largest measuring 150 × 140 mm, encased by the mesentery, small intestine, colon, and greater omentum. Additional tumors were identified on the anterior wall of the greater curvature of the stomach and in the left lateral segment of the liver, necessitating partial gastrectomy and left lateral hepatectomy along with tumor excision. Histopathological analysis confirmed myxoid liposarcoma in all resected specimens (Fig. 5).

**Figure 4.**
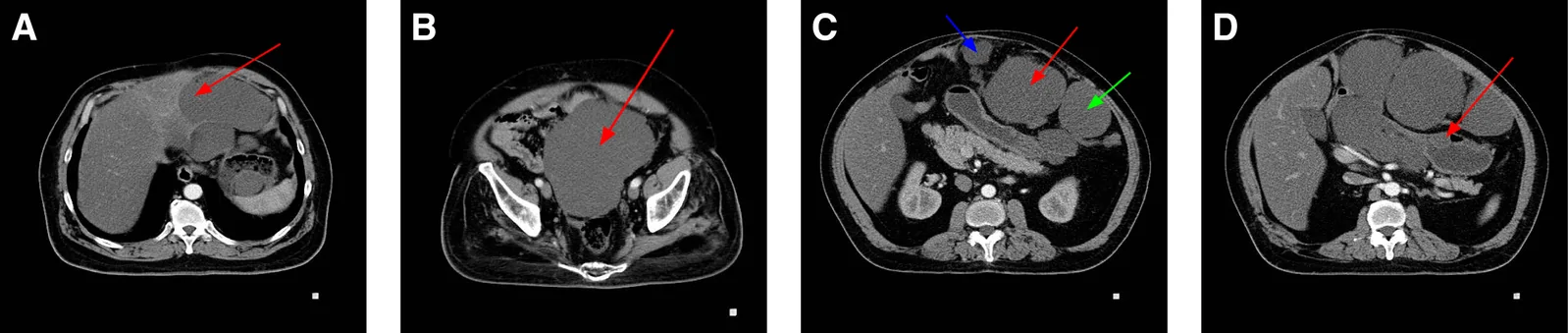
Contrast-enhanced computed tomography images of the abdomen and pelvis. (A) The tumor involves the liver and shows tight adhesion to the hepatic parenchyma. (B, C) Representative sections demonstrating different levels of the intra-abdominal mass. (D) Tumor arising from the gastric greater curvature.

**Figure 5.**
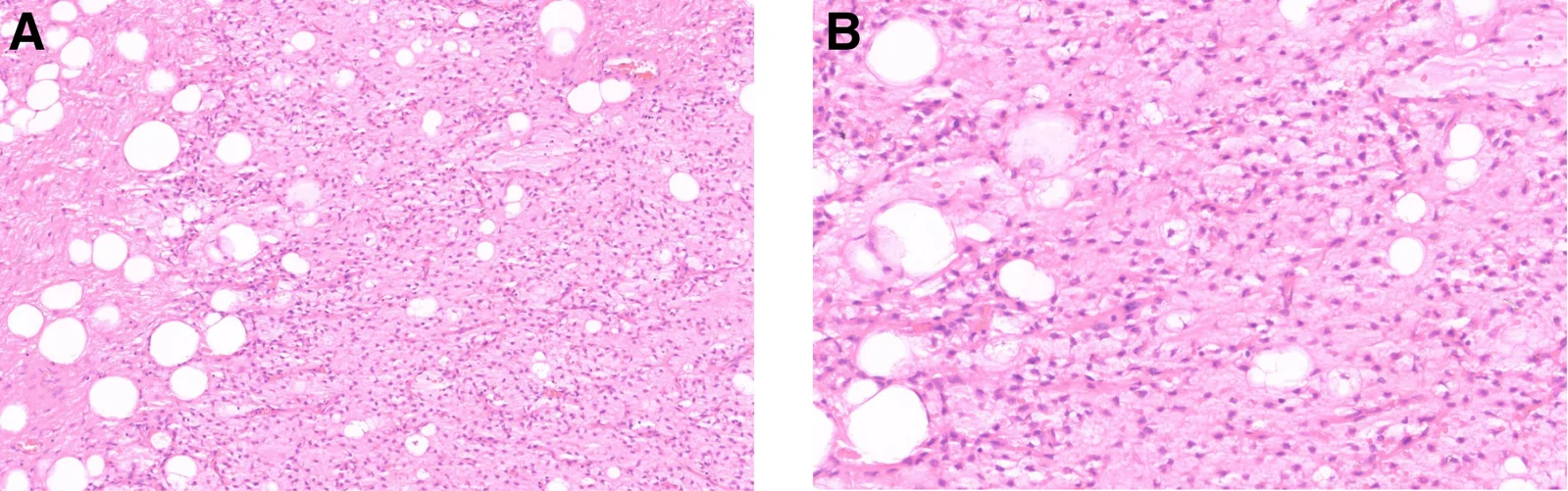
(A) A 10× microscopic image of the pathological specimen, showing a large number of fat cells. (B) A 20× microscopic image of the pathological specimen.

Following the repeat resection, the patient recovered uneventfully. At discharge, he was able to perform activities of daily living independently, tolerated a regular diet, and had normal bowel function without significant discomfort. He was referred to the oncology department for further management and was advised to undergo regular clinical and radiological surveillance. However, the patient did not adhere to the recommended follow-up schedule, and detailed long-term postoperative functional outcomes and surveillance data were therefore unavailable.

## 3. Discussion

Such a massive RPLS is extremely rare, with few cases reported in the English literature. To our knowledge, this represents an exceptionally large recurrent RPLS, with a high recurrence rate. Postoperative recurrence of liposarcoma typically occurs within 6 months to 2 years after initial resection.^[11]^ The likelihood of complete surgical resection is up to 80% for first-time recurrences but decreases to 60% to 70% for subsequent recurrences.^[8]^ Median survival after surgical resection has been reported as 83 months,^[12]^ with complete tumor excision associated with a mean survival of 8 years and a 5-year disease-free survival of 60%.^[13]^ Three-year disease-free survival is 73% for patients with negative margins compared with 43% for those with incomplete resection.^[14]^

In the present case, the patient experienced 4 recurrences. Achieving negative surgical margins through complete resection is critical to reducing recurrence risk and improving quality of life, although it remains technically challenging and heavily dependent on surgical expertise. Surgery may be combined with neoadjuvant systemic therapy or radiotherapy. Grosso et al retrospectively analyzed 51 liposarcoma cases,^[15]^ showing that perioperative chemotherapy did not significantly improve overall survival but effectively reduced local recurrence rates, and patients receiving surgery plus radiotherapy had higher 5-year survival than those undergoing surgery alone.^[6]^ These findings suggest that, although the roles of chemotherapy and radiotherapy are limited, they still hold substantial clinical value in reducing recurrence and improving survival. To date, no randomized controlled trials have definitively established the efficacy of chemotherapy or radiotherapy in liposarcoma, and further research is needed to optimize their use for patient benefit.

Several giant RPLSs of comparable or greater size have been reported. Xu et al described a tumor measuring 37 × 32 × 26 cm and weighing 21 kg,^[16]^ while Kanthala et al reported an 18.55 kg recurrent dedifferentiated RPLS.^[8]^ More recently, Sun et al reported a tumor measuring 55 × 30 × 18 cm and weighing 19.8 kg.^[17]^ In comparison, the tumor in the present case measured 43 × 32 × 23 cm and weighed approximately 20 kg, placing it within the upper range of giant RPLSs reported in the literature.

The distinctive feature of the present case lies not solely in the tumor size, but in the combination of an extreme tumor burden and a prolonged 13-year clinical course characterized by multiple recurrences and repeated surgical resections. Despite occupying almost the entire abdominal cavity and causing marked displacement of adjacent organs, the giant recurrent tumor remained surgically resectable, with preservation of the major abdominal organs apart from partial diaphragmatic resection. Nevertheless, multifocal abdominopelvic recurrence developed 18 months later and required further tumor excision combined with partial gastrectomy and left lateral hepatectomy. This clinical course illustrates both the feasibility of repeated surgical treatment in carefully selected patients and the progressively increasing complexity of surgery for recurrent disease. It also underscores the importance of multidisciplinary operative planning and structured long-term surveillance.

Nevertheless, several limitations should be acknowledged. First, postoperative surveillance was incomplete because the patient did not adhere to the recommended follow-up schedule, resulting in a lack of interval clinical and radiological data before recurrence was detected. Second, no adjuvant chemotherapy or radiotherapy was administered after the most recent resection, and therefore the potential benefit of postoperative multimodal treatment could not be evaluated in this case. Third, detailed information regarding the chemotherapy regimen, radiation dose, treatment fields, and therapeutic response following the earlier operation was unavailable because of the long interval since treatment and incomplete historical medical records. These limitations restrict a comprehensive assessment of treatment efficacy and long-term disease control.

## 4. Conclusion

Even exceptionally large recurrent RPLSs may remain surgically resectable after careful imaging assessment and multidisciplinary planning. Repeated surgery can provide symptom relief and disease control, but the high risk of multifocal recurrence underscores the need for individualized postoperative management and structured long-term surveillance.

## Acknowledgments

We would like to thank the surgical, pathology, and radiology teams for their valuable contributions to the diagnosis and management of this case. We also appreciate the support of our colleagues for their assistance in data collection and manuscript preparation.

## Author contributions

**Data curation:** Meng-wei Tu.

**Visualization:** Meng-wei Tu, Song Zhu.

**Writing – original draft:** Meng-wei Tu, Song Zhu.

**Formal analysis:** Song Zhu.

**Investigation:** Song Zhu.

**Funding acquisition:** Jian-bo Zhang.

**Writing – review & editing:** Jian-bo Zhang.
